# Dr. Kinglake, in Reply to Verax, on Gout

**Published:** 1808-07

**Authors:** Robert Kinglake

**Affiliations:** Taunton


I am, &c.

T. H.

*June*, 9, 1808.

---

### *To the Editors of the Medical and Physical Journal.*

Gentlemen,

YOUR correspondent, under the signature of *Verax*, has in your last Journal required my opinion on the observations he has therein published, respecting the use of topical cold in gout. I have no desire to resist the application, though it must be confessed, it would have had a stronger claim to my compliance, if the author had made the demand in his proper name. In correctly investigating a medical medical subject, too much of real philosophy should direct the inquiry to admit of regarding any consideration that did not tend to render the research successful.

The motives and conduct of your correspondent in subjecting the cooling treatment of gout to trial, appear to be worthy of any name or rank in the medical profession, the author therefore needed not the disguise of a fictitious signature.

The cases in which "*Verax*" witnessed the beneficial effects of the cooling treatment of gout are sufficient to demonstrate to him that gouty inflammation, like all other diseases, may be safely and advantageously resisted. But this your correspondent thinks can only be done under the circumstances of youth, health, and constitutional strength, which he describes to have existed in the patients to whom the remedy was administered under his direction. It would be superfluous in me, at this advanced stage of the inquiry, to restate the refutation which this opinion has received in the numerous cases published, in which, much diversity of age, temperament, and concomitant disease, advantageously underwent the full efficacy of topical cold. Desirable as it may be to the timid, philosophical as it may appear to the rational, to have the respective limits of the safety and danger of the practice precisely marked out, it is not yet in my power to define the description of cases in which it would be unsafe or hurtful. In my *additional cases* lately published on the subject, are offered some observations for consideration as to the *impolicy* of resorting to the treatment where existing visceral disease and advanced age may give occasion to equivocal co-incidences, and tend to embarrass the fair progress of the inquiry.

It has never been pretended that topical cold will at once reduce inflammatory heat, and cure an indurated and thickened state of the ligamentous and tendinous structure. It will be therefore found that the treatment in such cases is more likely to prove a palliating than a radical remedy. By stemming the progress of the mischief already done, it promises to obviate farther evil, and thus to afford an opportunity for gradual amendment, if not for an ultimate reparation of the damaged fabric.

In asserting that the treatment is applicable in some instances and not in others, the dilemma is incurred of proposing at best a precarious remedy. Its safety in that case must depend on the correctness of discrimination that may have been exerted, which must render its adoption on every occasion too ambiguous, too fearful, ever to be fully justified.

justified. If it be admitted that gout is a transient disease, that it has no fixed abode, that at one time by a salutary effort of nature it is in the great toe-joint, and at another, by morbid retrocession in the stomach, or any other vital organ, where is the authority, or where the boldness, that would venture on even an occasional trial of the influence of topical cold in that ailment?

Could it be imagined with "*Verax*" that those cases only of gout that are shielded by youth, vigour, and an unestablished state of the disease, are safely assailable by topical cold, what vindicable motive could be assigned for hazarding a dubious remedy against an affection that in most instances is supposed to prove beneficial to the general health?

The practical difficulty suggested by your correspondent respecting the application of the cooling treatment of gout, would appear to amount to no more than that, in certain cases in which the joints may not have been deeply injured by repeated and long continued paroxysms of gout, topical cold is directly and permanently curative, whilst it only temporarily alleviates the torture of joints inflamed, enlarged, and spoilt by the long standing and protracted ravages of the disease.

Deplenishing the inflamed vessels by drawing blood from the affected part with leeches, has not appeared in my experience, as it would seem to have done in that of "*Verax*," a previous requisite to the use of topical cold; it certainly may act as an auxiliary, but it is the aid which the usual efficacy of topical cold does not require, and which, therefore, unnecessarily diversifies the means of remedy. Indeed, the objection to it goes farther than its being superfluous; it may so far debilitate the inflamed vessels, as to lessen, rather than augment, (as "*Verax*" supposes) their re-active power, and thereby expose them to the risk of being chilled or benumbed by a free application of cold.

The extraordinary circumstances attending the case of the vintner, on which your correspondent has desired my opinion, deserves serious consideration. It will be found, I apprehend, on close examination, that the singular swelling of the genital parts alluded to, must have arisen from checked perspiration, induced, perhaps, by an undue use of topical cold during the three or four days it is stated to have been employed. Whether the inflammatory appearance on the elbow-joint was of a gouty nature, or resulted, as the patient himself imagined, from a sprain incurred in the exertion in which he was occupied, is not of much im-

 portance portance to determine, since it is well known that gouty inflammation, and sprain from external violence, reciprocally produce and suspend each other. They are diseases of the same structure, and subjected to the same laws in both the original and sympathetic action of their respective vital power.

It does not appear that the pain in the elbow and finger-joints had been subdued before the tumefaction took place in the genital parts; that occurrence, therefore, could not have resulted from a transference of the primary or gouty irritation; on the contrary, it is reasonable to conclude, that the continued violence of the gouty inflammation had induced symptoms of general irritation on the febrile state, said to have affected the system, and also the extraordinary effusion of water in the cellular structure of the external parts of generation. The tumour that presented, is stated to have assumed a livid hue; this must, it may be presumed, have arisen from an interruption given to the refluence of venous blood rather than from approaching gangrene.

It is not, indeed, observed, that severe inflammatory pain had preceded the appearance of the enlargement mentioned. This swelling, from its resembling the aspect exhibited by those parts on which it occurred, when distended by the effusion of urine into their cellular texture, was probably œdematous, and does not appear to have had any intelligible connection with gouty inflammation in either the stationed or shifting character of that disease. I know of no similar occurrence pending a paroxysm of gout; but an œdematous tumefaction of the penis and scrotum sometimes happens during the progress of chronic and towards the close of acute diseases; several instances of which have fallen under my own observation.

The cooling treatment of gout is either salutary or noxious, but the one condition or the other of its power must stand distinctly on its own efficacy; it must not be incumbered and obscured by irrelevant accidents, because they present simultaneously. Acuteness in medical observation is never more usefully or signally evinced, than in duly distinguishing and arranging morbid occurrences, so that causes and effects may maintain a clear and undisturbed connection. Time is no accurate test of the nature of morbid co-incidences; *post hoc ergo propter hoc*, is fallacious logic on medical as well as on all other occasions. The quality of an occurrence is a surer guide in search of hindered Nature than the period when it happened; thus, in in the case in question, would it be reasonable to infer relationship or necessary connection between an inflammatoy affection on the elbow-joint, and an œdematous swelling from serous affusion on the penis and scrotum? The latter may, indeed, be a distant result of the former, but could not be involved as a direct consequence.

Your correspondent, under the assumed name of *Verax*, is entitled to my respectful consideration for the candid trial he has given the cooling treatment of gout. He has evidently entered on the practice with the most benevolent intention, and with that freedom from popular prejudice which enabled him to be sufficiently confident in its safety to submit it to experiment. His experience of its efficacy, as far as it has gone, appears to have been favourable enough, at once to justify the trial he has made, and to encourage its farther continuance. If no case more adverse to its success than that of the vintner is known to him, he surely may venture to resume his last confidence in its safety, and to prosecute the practice with unabated expectation of advantage from its employ.

Whilst on the subject of the cooling treatment of gout, I think it incumbent on me to aver, that it continues to afford in this neighbourhood, in as far as I have had an opportunity of learning its effects, all the benefit that can reasonably be desired, or that its advocates ever promised. Were it in my power to state any circumstance that militated against the principle on which the practice is founded, it would be disregarding the benevolence of my motive in originally proposing it for the relief of the sufferings of mankind, if I did not unreservedly make such objection known. The true merits of the practice will be best ascertained by neglecting no opportunity of faithfully noticing and recording its occurring defects.

I am, &c.

ROBERT KINGLAKE.

*Taunton, June 9, 1808.*

